# Doxycycline Attenuated Ethanol-Induced Inflammaging in Endothelial Cells: Implications in Alcohol-Mediated Vascular Diseases

**DOI:** 10.3390/antiox11122413

**Published:** 2022-12-07

**Authors:** Xuanchen Li, Dilaware Khan, Majeed Rana, Daniel Hänggi, Sajjad Muhammad

**Affiliations:** 1 Department of Neurosurgery, Medical Faculty, Heinrich-Heine-University, Moorenstrasse 5, 40225 Düsseldorf, Germany; 2 Department of Oral and Maxillofacial Surgery, Medical Faculty, Heinrich-Heine-University, Moorenstrasse 5, 40225 Düsseldorf, Germany; 3 Department of Neurosurgery, University Hospital Helsinki, Topeliuksenkatu 5, 00260 Helsinki, Finland

**Keywords:** ethanol, HUVECs, inflammaging, telomere shortening, accelerated aging, cellular senescence, doxycycline, mTOR, NFκ-B

## Abstract

Excess alcohol consumption is a potential risk factor for cardiovascular diseases and is linked to accelerated aging. Drug discovery to reduce toxic cellular events of alcohol is required. Here, we investigated the effects of ethanol on human umbilical vein endothelial cells (HUVECs) and explored if doxycycline attenuates ethanol-mediated molecular events in endothelial cells. Initially, a drug screening using a panel of 170 drugs was performed, and doxycycline was selected for further experiments. HUVECs were treated with different concentrations (300 mM and 400 mM) of ethanol with or without doxycycline (10 µg/mL). Telomere length was quantified as telomere to single-copy gene (T/S) ratio. Telomere length and the mRNA expression were quantified by qRT-PCR, and protein level was analyzed by Western blot (WB). Ethanol treatment accelerated cellular aging, and doxycycline treatment recovered telomere length. Pathway analysis showed that doxycycline inhibited mTOR and NFκ-B activation. Doxycycline restored the expression of aging-associated proteins, including lamin b1 and DNA repair proteins KU70 and KU80. Doxycycline reduced senescence and senescence-associated secretory phenotype (SASP) in ethanol-treated HUVECs. In conclusion, we report that ethanol-induced inflammation and aging in HUVECs were ameliorated by doxycycline.

## 1. Introduction

Aging is strongly associated with cardiovascular diseases. With increasing age, the risk of multiple cardiovascular diseases increases. The senescence of vascular cells is associated with age-related cardiovascular diseases [1,2,3]. Senescence is a state of irreversible growth arrest, which can be either replicative senescence or stress-induced premature senescence. Replicative senescence results from telomere shortening. With each cell division, telomeres shorten, and when these telomeres are shortened to a certain degree, cells become replicative senescent [4]. Stress-induced premature senescence can be induced by various stress signals, including telomere shortening and DNA damage by oxidative stress, metabolic stress, oncogenic activation, and constitutive activation of mitogenic stimuli [1,5].

The senescence and dysfunction of vascular endothelial cells play a vital role in vascular aging, which results in the initiation, progress, and advancement of cardiovascular diseases [1]. The senescence of endothelial cells is a potential link between aging and inflammation [2]. Senescent and dysfunctional endothelial cells actively express and release pro-inflammatory cytokines, chemokines, adhesion molecules, and matrix metalloproteinases (MMPs) [1,5,6,7,8], called senescent-associated secretory phenotype (SASP). Chronic sterile inflammation can enhance this process and consequently promotes tissue remodelling [1,8]. Clinical and experimental studies suggest that chronic, sterile, and low-grade inflammation is a sign of the aging process, namely inflammaging [2]. With increasing age, the pro-inflammatory shift in the gene expression of vascular endothelial and smooth muscle cells (SMCs) forms a pro-inflammatory microenvironment in the vascular wall, which consequently contributes to vascular diseases [2]. Senescent cells have been found in atherosclerotic tissue, and the progression of atherosclerosis could be prevented by eliminating senescent cells [2,9]. Animal studies have shown that the elimination of senescent cells provided protection against age-related diseases and increased life span [5]. Risk factors such as oxidative stress, alcohol abuse, smoking, obesity, diabetes, and aging can induce endothelial senescence and dysfunction, which contributes to atherosclerosis, arterial stiffness, and hypertension leading to cardiovascular diseases [1,2,8,9]. According to World Health Organization, an estimated 2.3 billion people are current alcohol drinkers, and 3 million people died as a result of the harmful use of alcohol in 2016 alone. Alcohol abuse is one of the most potentially acquired risk factors for cardiovascular diseases, and state-of-the-art research supports the notion that alcohol abuse accelerates aging [10,11]. However, hitherto the direct effect of alcohol on cellular aging and the underlying mechanisms have not been investigated.

Doxycycline is a broad-spectrum antibiotic belonging to the tetracycline class, and it has an anti-inflammatory effect. It has been reported to inhibit the activation of NF-κB [12,13]. Both NF-κB and mTOR pathways play an important role in inflammation and aging [7,14,15,16,17,18,19]. Inhibition of NF-κB and mTOR pathways curtails inflammation, reduces cellular senescence and delays aging [7,14,15,16,17,18,19].

This is the first report showing that ethanol accelerated cellular aging and senescence in HUVECs. Doxycycline treatment reduced ethanol-enhanced aging and cellular senescence by inhibiting NFκ-B activation and the mTOR pathway.

## 2. Methods

### 2.1. Cell Culture

HUVECs were obtained from Promocell (Heidelberg, Germany) and maintained in the medium consisting of endothelial cell medium (C-22010, Promocell, Heidelberg, Germany) supplemented with endothelial growth factors (C-39215, Promocell, Heidelberg, Germany) at 37 °C in a 95% humidified atmosphere containing 5% CO_2_. Cells were seeded in the T75 cell culture flask after thawing. When the cells were 90% confluence, they were incubated with trypsin for 4 min at 37 °C, and then cells were seeded in 10 cm cell culture plate, 5000 cells/cm^2^. For experiments, the endothelial cells were used at passage 7 and were seeded in 6-well plates. After 24 h, the medium was changed, and the new medium containing either ethanol (300 mM or 400 mM), doxycycline (10 µg/mL), or 400 mM ethanol combined with doxycycline (10 µg/mL) was added to the culture; the medium was changed after two days. Doxycycline was purchased from Sigma-Aldrich (D1822).

### 2.2. Telomere Length Measurement

In the first experiment, the endothelial cells were treated with an endothelial cell medium containing either 300 mM or 400 mM ethanol. In the second experiment, the endothelial cell medium was supplemented with either 400 mM ethanol or 400 mM ethanol combined with doxycycline (10 µg/mL). The endothelial cell medium alone was used for the control group. The medium was changed after two days. After four days of treatment, DNA was extracted from cell pellets using innuPREP DNA Mini Kit (845-KS-1042050, Analytik Jena, Jena, Germany). For telomere length measurement, 1 ng DNA was utilized. For qPCR the primers (Table S2) telomere (TEL) and single-copy gene interferon beta 1 (IFNB1) were used as previously described [20]. Quantitative PCR was performed with AceQ SYBR qPCR Master Mix (Q111-03, Vayzme, Nanjing, China) on Bio-Rad with an initial denaturation of 95 °C for 10 min, followed by 40 cycles of 95 °C for 15 s and 60 °C for 1 min, followed by melting curve. The relative telomere length was calculated as telomere to single copy-gene (IFNB1) ratio. The T/S was quantified using the comparative ΔCT method. All experiments were performed with three biological triplicates, and each biological replicate had three technical triplicates.

### 2.3. CellTiter-Glo (CTG) Assay

For the CTG assay, the cells were seeded in 96-well plate (5000 cells/cm^2^). The medium was changed the next day with a new medium alone (for control) or medium containing ethanol (200 mM, 300 mM, 400 mM, 600 mM, and 800 mM), doxycycline (1 µM, 10 µM, 50 µM, and 100 µM) and combination of ethanol (400 mM) and doxycycline (10 µM). The medium was changed after two days. The readout of the cell survival was performed using luminescence-based CTG assay (Promega, Walldorf, Germany) according to the manufacturer’s instructions, except that the reaction agent was diluted in 1:1 with PBS. The CTG assay was performed immediately after changing the medium on day 0 and on day 1, day 2, and day 4.

### 2.4. Western Blot

For protein analysis, endothelial cells were treated with endothelial cell medium supplemented with either 400 mM ethanol, doxycycline (10 µg/mL), or 400 mM ethanol combined with doxycycline (10 µg/mL). For the control group, the endothelial cells were treated with endothelial cell medium alone. After 24 h of treatment, the total protein was extracted using RIPA Buffer, then determined calorimetrically using the DC Protein Assay Kit (500-0116, Bio-Rad, Hercules, CA, USA), according to the manufacturer’s instructions, and measured with the Paradigm micro-plate reader. Total protein (25 µg, in reducing conditions) was loaded on 12% sodium dodecyl sulfate-polyacrylamide gel and run at 60 volt for 20 min, then continued with 110 Volt for 30–60 min, which was further transferred onto a polyvinylidene difluoride membrane at 250 mA for 120 min. The non-specific binding was blocked with 5% skim milk (0.05% TBST) for 1 h. The membranes were incubated with primary antibodies (as reported in Supplementary Table S1) overnight at 4 °C on a shaking platform. The membranes were washed thrice for 10 min with TBST and then incubated with secondary antibodies (Table S1) for 1 h at RT. All antibodies were diluted in the blocking solution containing either 5% bovine serum albumin (BSA) for phosphorylated proteins or 5% skim milk for the rest of the non-phosphorylated proteins. The antibodies were diluted in Tris-buffered saline with Tween20 (TBST). The densitometry was calculated with NIH-Image J (National Institutes of Health, Bethesda, MD, USA) by correcting for β-actin. All WBs were performed with three biological triplicates.

### 2.5. Quantitative PCR

For quantitative PCR (qPCR), total RNA was extracted from endothelial cells treated with either endothelial cell medium alone (control group) or endothelial cell medium containing either 400 mM ethanol or 400 mM ethanol combined with doxycycline (10 µg/mL) for 24 h. The Nucleo Spin RNA kit (740955.50, MACHEREY-NAGEL, Düren, Germany) was utilized to extract total RNA following the manufacturer’s instructions. RNA (1.2 µg) was utilized to reverse transcribe with M-MLV Reverse Transcriptase kit (M1701, Promega), Random Hexamer Primers (48190011, Thermo Fisher, Waltham, MA, USA), and RiboLock RNase Inhibitor (EO0384, Thermo Fisher, Waltham, MA, USA). qPCR was performed with AceQ SYBR qPCR Master Mix (Q111-03, Vayzme, Nanjing, China) on Bio-Rad with an initial denaturation of 95 °C for 8 min, followed by 45 cycles of 95 °C for 15 s, 58.9 °C for 30 s, and 72 °C for 30 s, followed by melting curve. The relative mRNA expressions were calculated after normalizing them to β-actin expression. The primer sequences are listed in Table S2. Relative mRNA expression was quantified using the comparative ΔCT method. All experiments were performed with three biological triplicates, and each biological replicate had three technical triplicates. The data are shown for one biological replicate.

### 2.6. Migration Assay

HUVECs were seeded in a 6-well plate. Once the cells were more than 95% confluence, a 10 μL sterile pipette tip was used to make a scratch on the monolayer of endothelial cells. After the scratch, the endothelial cells were washed 3 times with PBS, and a new endothelial cell medium containing either 400 mM ethanol or 400 mM ethanol combined with doxycycline (10 µg/mL) was added to the culture. For the control group, the endothelial cells were treated with endothelial cell medium alone. At 0 h, 6 h, 12 h, and 24 h following the scratch assay, images of cell migration in three randomly selected fields were taken for each well using an optical microscope. Finally, the area of the wound was measured by Image J. All experiments were performed with three biological triplicates, and each biological replicate had three technical triplicates.

### 2.7. β-Gal Staining

β-Gal staining was detected using a Senescence Cells Histochemical Staining Kit (GALS, Sigma, St. Louis, MO, USA), following manufacturer instructions. The endothelial cells were treated with endothelial cell medium alone (control group) or endothelial cell medium supplemented with either 400 mM ethanol or 400 mM ethanol combined with doxycycline (10 µg/mL). The medium was changed after two days. After 4 days, the cells were fixed in the fixation buffer provided with the Senescence Cells Histochemical Staining Kit. The fixed samples were stained with fresh solution for SA-beta-galactosidase activity at 37 °C for 7 h, followed by aspiration with the staining solution. After that, the cells were overlaid with a 70% glycerol solution and stored at 4 °C. The images were taken with an optical microscope. Finally, the number of stained cells was counted using Image J. The experiment was performed with three biological triplicates.

### 2.8. Immunofluorescence Staining

The cells were seeded in a 96-well plate (5000 cells/cm^2^). The medium was changed the next day with a new medium alone (for control) or a medium containing ethanol (400 mM), doxycycline (10 µM), and a combination of ethanol (400 mM) and doxycycline (10 µM). After two hours of incubation, immunofluorescence staining was performed. The cells were washed 3 times with PBS and fixed with 4% paraformaldehyde for 15 min, permeabilized with 0.2% Triton™ X-100 for 10 min, and blocked with 5% BSA for 1 h at RT. The cells were incubated overnight at 4 °C with primary antibody 8-OHDG (1:500, Cat. No.: BSS-BS-1278R, BIOSS, Woburn, MA, USA). The next day, the cells were washed three times and then labeled with Secondary antibody (1:1000, Alexa Fluor 488, Cat. No.: ab150077) for 60 min at RT. Nuclei were stained with SlowFade^®^ Gold Antifade Mountant with DAPI (Product # S36938) for 10 min. The images were captured at 20× magnification.

### 2.9. Statistical Analysis

Student’s *t*-test was performed to compare two groups, and one-way ANOVA followed by Tukey’s test was used to analyze more than two groups. The level of significance was set at * *p* < 0.05.

## 3. Results

### 3.1. Ethanol-Enhanced Molecular Aging in Endothelial Cells

**Figure 1:**
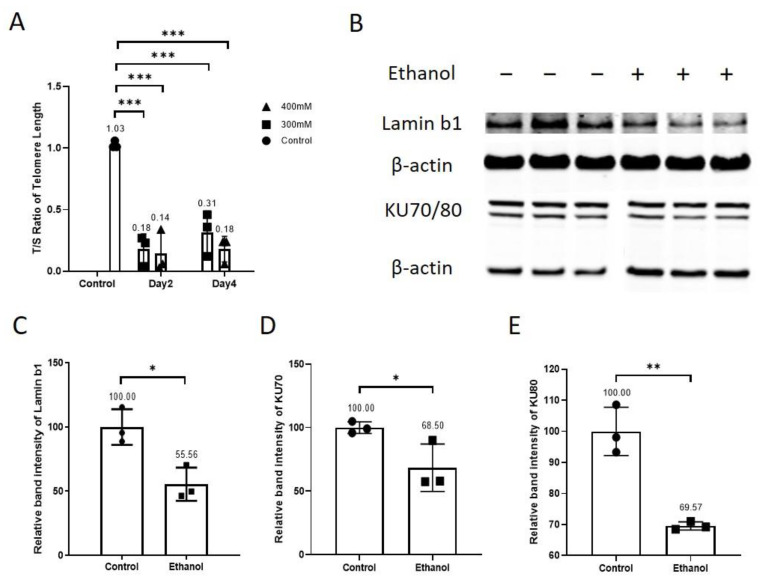
Ethanol accelerated molecular aging in endothelial cells. (**A**) The telomere length was shortened after ethanol (300 mM and 400 mM) treatment in HUVECs. (**B**) WB showing protein expression of lamin b1, KU70 and KU80 in HUVECs 24 h after ethanol treatment (400 mM). Ethanol exposure reduced the relative protein expression of (**C**) lamin b1, (**D**) KU70, and (**E**) KU80. β-actin was used as a loading control. Data are the mean of three independent biological triplicates. Student’s *t*-test was performed to compare two groups, and one-way ANOVA followed by Tukey’s test was used to analyze more than two groups. Error bars represent the SD (*** *p* < 0.001, ** *p* < 0.01, and * *p* < 0.05).

Here, we investigated the effects of two different concentrations (300 mM and 400 mM) of ethanol on the telomere length of endothelial cells. The endothelial cells were treated with an endothelial cell medium containing either 300 mM or 400 mM ethanol. The endothelial cell medium without ethanol was used for the control group. Telomere length was quantified as T/S. The telomere length was shortened by more than 50% in ethanol-treated HUVECs as compared to that in the untreated control (Control = 1.03 ± 0.03 T/S, 300 mM ethanol day 2 = 0.18 ± 0.13 T/S, 400 mM ethanol day 2 = 0.14 ± 0.17 T/S, 300 mM ethanol day 4 = 0.32 ± 0.17 T/S, 400 mM ethanol day 4 = 0.18 ± 0.10 T/S, n = 3, *** *p* < 0.001, Figure 1A). Next, we investigated the protein expression of aging-related protein lamin-b1 and DNA repair proteins KU70 and KU80 after 24 h of 400 mM ethanol treatment. Protein analysis showed a remarkably reduced relative protein expression of lamin b1 24 h after ethanol exposure (Control = 100 ± 13.85, ethanol = 55.56 ± 12.95, n = 3, * *p* < 0.05, Figure 1B,C), indicating a disruption in nuclear structure. The ethanol treatment decreased the relative protein expression of DNA repair protein KU70 (Control = 100 ± 4.52, ethanol = 68.50 ± 18.69, n = 3, * *p* < 0.05, Figure 1B,D), and KU80 (Control = 100 ± 7.77, ethanol = 69.57 ± 1.3, n = 3, ** *p* < 0.01, Figure 1B,E), showing the diminished capacity of DNA repair. Ethanol treatment accelerated aging by shortening telomere length and reducing the relative protein expression of aging-associated protein lamin b1 and DNA repair proteins KU70 and KU80.

### 3.2. Doxycycline Reduced the Toxic Effects of Ethanol

To test the toxicity of ethanol, doxycycline and a combination of ethanol and doxycycline, we performed CellTiter-Glo (CTG) assay. The cells were treated with different concentrations of ethanol, doxycycline, and combined ethanol (400 mM) and doxycycline (10 µM). The medium was changed after two days. The CTG assay was performed on day 0, day 1, day 2, and day 4. On day 0, no difference was observed between the groups (Day 0: Control = 47.54 ± 8.84%, ethanol (200 mM) = 49.26 ± 9.34%, ethanol (300 mM) = 48.05 ± 8.93%, ethanol (400 mM) = 50.22 ± 15.67%, ethanol (600 mM) = 51.76 ± 10.25%, ethanol (800 mM) = 54.26 ± 9.97%, doxycycline (1 µM) = 48.32 ± 11.54%, doxycycline (10 µM) = 46.72 ± 10.73%, doxycycline (50 µM) = 46.71 ± 12.83%, doxycycline (100 µM) = 48.30 ± 11.91%, ethanol + doxycycline = 43.12 ± 9.23%, *p* > 0.05, n = 3, Supplementary Figure S1). On day 1 control did not show the difference in all concentrations of ethanol. Doxycycline (50 µM and 100 µM) showed improved viability than control and ethanol (300 mM). Ethanol 400 mM showed reduced growth than doxycycline (10 µM, 50 µM, and 100 µM) and the combination of ethanol (400 mM) and doxycycline (10 µM). Ethanol (600 mM and 800 mM) showed reduced growth than ethanol (400 mM) and doxycycline (10 µM) combined, and all concentrations of doxycycline used (Day 1: Control = 49.62 ± 3.11%, ethanol (200 mM) = 51.30 ± 2.37%, ethanol (300 mM) = 46.31 ± 1.24%, ethanol (400 mM) = 44.41 ± 1.22%, ethanol (600 mM) = 40.58 ± 1.08%, ethanol (800 mM) = 40.88 ± 3.10%, doxycycline (1 µM) = 51.97 ± 2.81%, doxycycline (10 µM) = 54.23 ± 3.58%, doxycycline (50 µM) = 59.25 ± 3.32%, doxycycline (100 µM) = 61.01 ± 4.36%, ethanol + doxycycline = 54.09 ± 1.69%, *p* < 0.05, n = 3, Supplementary Figure S1). On day 2, ethanol (300 mM, 400 mM, 600 mM, and 800 mM) showed decreased growth than the control, doxycycline (100 µM), and the combination of ethanol (400 mM) and doxycycline (10 µM) (Day 2: Control = 66.32 ± 1.74%, ethanol (200 mM) = 61.34 ± 3.44%, ethanol (300 mM) = 59.32 ± 2.10%, ethanol (400 mM) = 58.53 ± 1.84%, ethanol (600 mM) = 59.21 ± 1.58%, ethanol (800 mM) = 59.04 ± 2.32%, doxycycline (1 µM) = 64.95 ± 2.30%, doxycycline (10 µM) = 64.90 ± 1.35%, doxycycline (50 µM) = 63.44 ± 1.32%, doxycycline (100 µM) = 67.77 ± 3.47%, ethanol + doxycycline = 66.75 ± 3.36%, *p* < 0.05, n = 3, Supplementary Figure S1). On day 4, ethanol (300 mM, 400 mM, 600 mM, and 800 mM) and doxycycline (1 µM and 100 µM) showed impeded growth than the control. Ethanol (300 mM) showed reduced growth than doxycycline (10 µM, 50 µM) and a combination of ethanol (400 mM) and doxycycline (10 µM). Ethanol (400 mM) showed decreased growth than doxycycline (1 µM, 10 µM, and 50 µM) and a combination of ethanol (400 mM, 600 mM, and 800 mM) and doxycycline (10 µM) (Day 4: Control = 100.00 ± 0.00%, ethanol (200 mM) = 95.90 ± 2.14%, ethanol (300 mM) = 82.48 ± 2.51%, ethanol (400 mM) = 80.75 ± 2.50%, ethanol (600 mM) = 76.04 ± 2.10%, ethanol (800 mM) = 79.77 ± 3.19%, doxycycline (1 µM) = 88.81 ± 1.37%, doxycycline (10 µM) = 98.61 ± 1.42%, doxycycline (50 µM) = 95.11 ± 5.13%, doxycycline (100 µM) = 77.00 ± 2.11%, ethanol + doxycycline = 104.15 ± 3.32%, *p* < 0.05, n = 3, Supplementary Figure S1). For statistical analysis, ANOVA was used. The experiment was performed with three biological triplicates. Doxycycline 10 µM, the concentration that had no cytotoxic effects was used for further experiments.

### 3.3. Doxycycline Reduced Ethanol-Accelerated Aging

**Figure 2:**
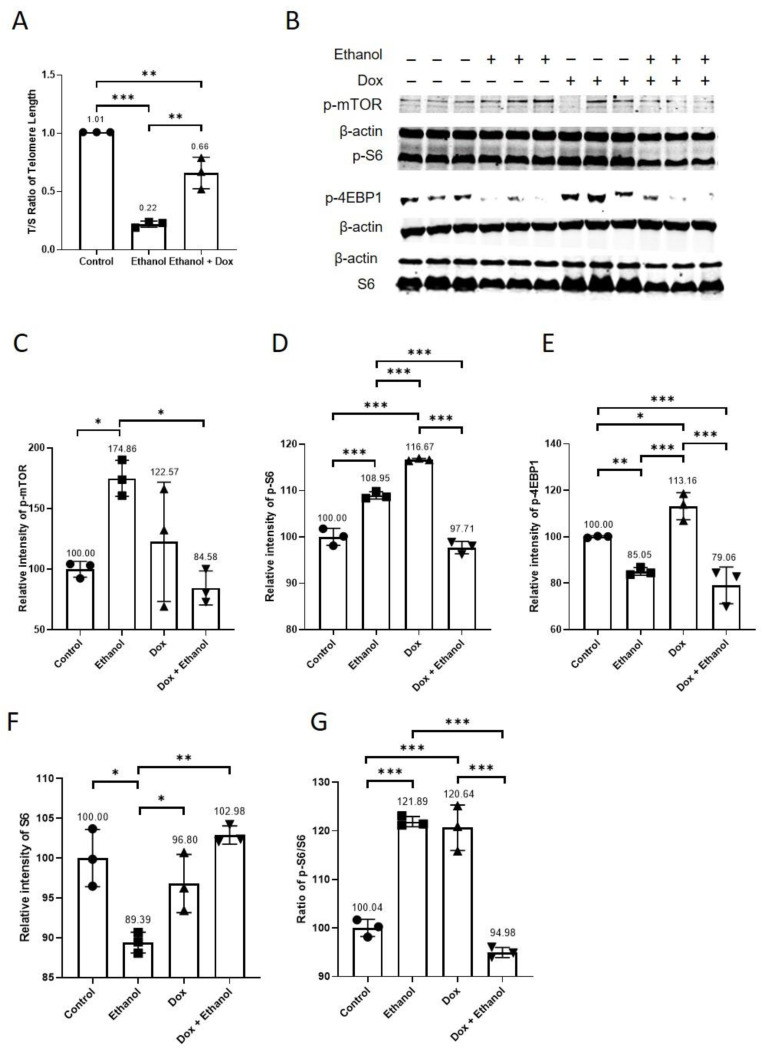
Doxycycline decreased ethanol-accelerated aging and inhibited ethanol-induced mTOR activation in HUVECs. HUVECs were treated with ethanol (400 mM), doxycycline (10 µM), and combination of ethanol (400 mM) and doxycycline (10 µM) for 24 h. The endothelial cell medium alone was used for control. (**A**) Doxycycline reduced ethanol-induced shortening of telomere length. (**B**) WB showing protein expression of mTOR pathway proteins. Doxycycline inhibited ethanol-induced activation of (**C**) mTOR and mTOR downstream signaling molecule (**D**) S6. (**E**) Doxycycline did not affect the relative protein expression of p-4EBP1 in ethanol-treated HUVECs. (**F**) Ethanol alone decreased relative protein expression of S6, and doxycycline combined with ethanol increased the relative protein expression of S6 more than only ethanol-treated HUVECs. (**G**) Both doxycycline and ethanol increased p-S6/S6 more than control and combination of doxycycline and ethanol decreased p-S6/S6 more than ethanol alone and doxycycline alone. β-actin was used as a loading control. Data are the mean of three independent biological triplicates. One-way ANOVA followed by Tukey’s test was used to analyze more than two groups. Error bars represent the SD (*** *p* < 0.001, ** *p* < 0.01, and * *p* < 0.05).

HUVECs were treated with either 400 mM ethanol or 400 mM ethanol combined with doxycycline (10 µg/mL) in endothelial cell medium. The control group was treated with endothelial cell medium alone. The medium was changed after two days. On day 4, we collected the cell pellets and extracted DNA. Telomere length quantification showed that doxycycline treatment ameliorated ethanol-accelerated telomere shortening in HUVECs (Control = 1.01 ± 0.00 T/S, 400 mM ethanol = 0.22 ± 0.03 T/S, 400 mM ethanol + doxycycline = 0.66 ± 0.14 T/S, n = 3, *** *p* < 0.001, ** *p* < 0.01 Figure 2A). It is known that the activation of the mTOR pathway plays an important role in aging [7,18,19], therefore we investigated the effects of ethanol, doxycycline, and ethanol combined with doxycycline on the activation of mTOR and its downstream targets. For protein analysis, we treated the endothelial cells with endothelial cell medium supplemented with either 400 mM ethanol, 10 µg/mL doxycycline or 400 mM ethanol in combination with 10 µg/mL doxycycline for 24 h. The endothelial cell medium alone was used for the control group. Doxycycline inhibited ethanol-induced activation of mTOR, relative protein expression of p-mTOR (Control = 100 ± 6.49, ethanol = 174.87 ± 14.8, doxycycline = 122.57 ± 49.15, ethanol + doxycycline = 84.58 ± 14.05, n = 3,* *p* < 0.05, Figure 2B,C). Both ethanol and doxycycline increased the relative protein expression of mTOR downstream signaling molecule p-S6 but ethanol and doxycycline in combination reduced its relative protein expression (Control = 100 ± 1.83, ethanol = 108.95 ± 0.78, doxycycline = 116.67 ± 0.27, ethanol + doxycycline = 97.71 ± 1.35, n = 3, *** *p* < 0.001, Figure 2B,D). Ethanol decreased relative protein expression of S6 and doxycycline increased the relative expression of S6 protein in ethanol treated HUVECs (Control = 100 ± 3.59, ethanol = 89.39 ± 1.31, doxycycline = 96.80 ± 3.66, ethanol + doxycycline = 102.88 ± 1.15, n = 3, ** *p* < 0.01, * *p* < 0.05, Figure 2F). Both ethanol and doxycycline increased the ratio of p-S6/S6, but the combination of ethanol and doxycycline treatment decreased p-S6/S6 in HUVECs (Control = 100 ± 1.77, ethanol = 121.89 ± 1.05, doxycycline = 120.64 ± 4.68, ethanol + doxycycline = 94.98 ± 1.04, n = 3, ** *p* < 0.01, * *p* < 0.05, Figure 2G). Doxycycline alone increased the relative protein expression of *p*-4EBP1, but it did not affect its relative protein expression in ethanol-treated HUVECs (Control = 100. ± 0.40, ethanol = 85.05 ± 1.66, doxycycline = 113.16 ± 5.83, ethanol + doxycycline = 79.06 ± 7.94, n = 3, *** *p* < 0.001, ** *p* < 0.01, and * *p* < 0.05; Figure 2B,E). Doxycycline decreased telomere shortening and inhibited activation of mTOR and its downstream target S6 in ethanol-treated HUVECs.

To investigate ethanol-induced oxidative stress, we performed immunofluorescence staining for oxidative stress marker 8-OHDG. HUVECs were treated with ethanol (400 mM), doxycycline (10 µM), and a combination of ethanol (400 mM) and doxycycline (10 µM) for 2 h. The endothelial cell medium alone was used for control. Ethanol increased oxidative stress, which was ameliorated by doxycycline (Control = 0.58 ± 0.53, Ethanol = 90.52 ± 2.65, doxycycline = 3.84 ± 4.94, ethanol ± doxycycline = 12.37 ± 15.51, *p* < 0.001, Supplementary Figure S2). The experiment was performed with three biological triplicates, and each biological replicate had three technical triplicates. For statistical analysis, ANOVA was used (*** *p* < 0.001).

### 3.4. Doxycycline Inhibited Ethanol-Induced NFκ-B Activation

**Figure 3:**
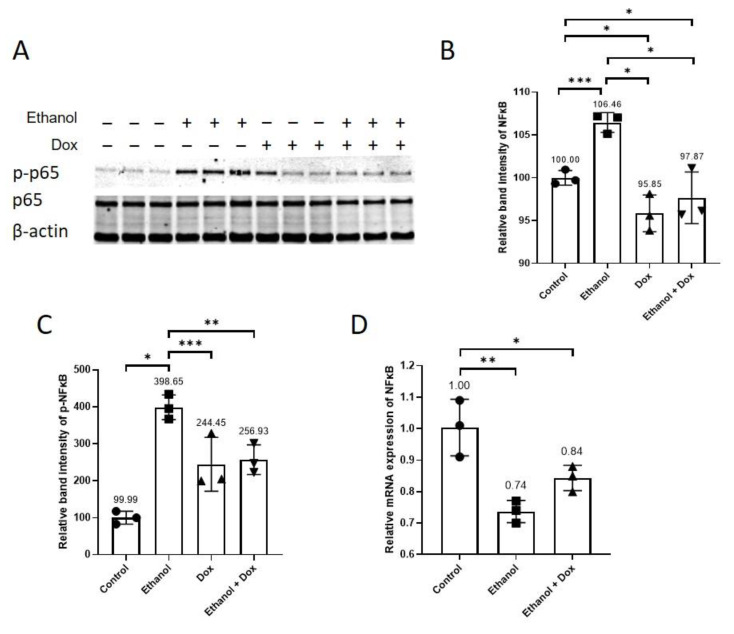
Doxycycline suppressed ethanol-induced NFκ-B activation in HUVECs. HUVECs were treated with ethanol (400 mM), doxycycline (10 µM), and combination of ethanol (400 mM) and doxycycline (10 µM) for 24 h. The endothelial cell medium alone was used for control. (**A**) WB showing the protein expression of p65 and p-p65. Doxycycline abated ethanol-induced relative protein expression of (**B**) p65 and (**C**) p-p65 in HUVECs. (**D**) Both ethanol (400 mM) and ethanol (400 mM) combined with doxycycline reduced mRNA expression of NF-κB. β-actin was used as a loading control. qPCR data are the mean of three technical triplicates, and WB data are the mean of three independent biological triplicates. One-way ANOVA followed by Tukey’s test was used to analyze more than two groups. Error bars represent the SD (*** *p* < 0.001, ** *p* < 0.01, * *p* < 0.05).

NFκ-B is known to play an important role in inflammation, aging and cellular senescence [14,15]. Ethanol can activate NFκ-B [21,22,23], and p-mTOR has been shown to activate NFκ-B [6]. Doxycycline reduced relative protein expression of NFκ-B p65 subunit in ethanol-treated HUVECs (Control = 100 ± 0.86, ethanol = 106.46 ± 1.14, doxycycline = 95.85 ± 2.15, ethanol + doxycycline = 97.67 ± 3.01, n = 3, *** *p* < 0.001, ** *p* < 0.01, * *p* < 0.05 Figure 3A,B). Ethanol increased activation of NFκ-B and doxycycline attenuated ethanol-induced NFκ-B activation in HUVECs, relative protein expression of p-p65 subunit of NFκ-B (Control = 100 ± 17.4, ethanol = 398.65 ± 33.54, doxycycline = 244.45 ± 72.76, ethanol + doxycycline = 256.93 ± 39.90, n = 3, ** *p* < 0.01, * *p* < 0.05, Figure 3A,B). Both ethanol and ethanol combined with doxycycline reduced mRNA expression of NF-κB (Control = 1.0 ± 0.09, ethanol = 0.74 ± 0.04, ethanol + doxycycline = 0.84 ± 0.04, ** *p* < 0.01, * *p* < 0.05, Figure 3D). Doxycycline impeded NFκ-B activation in ethanol-treated HUVECs.

### 3.5. Doxycycline Recovered the Relative Protein Expression of Aging-Related Biomarkers in Ethanol-Treated HUVECs

**Figure 4:**
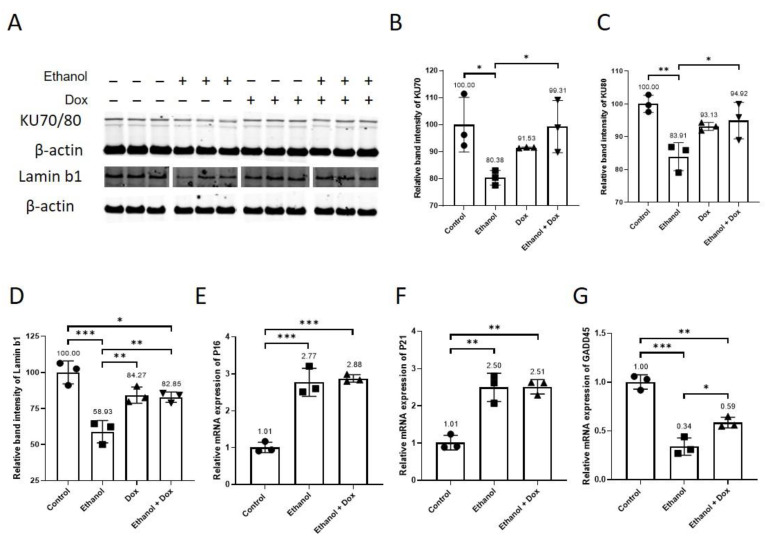
Doxycycline restored the relative protein expression of aging biomarkers. HUVECs were treated with ethanol (400 mM), doxycycline (10 µM), and combination of ethanol (400 mM) and doxycycline (10 µM) for 24 h. The endothelial cell medium alone was used for control. (**A**) WB showing protein expression of aging-associated biomarker lamin b1 and DNA repair proteins KU70 and KU80. Doxycycline recovered the relative protein expression of (**B**) KU70, (**C**) KU80 and (**D**) lamin b1 in ethanol-treated HUVECs. Doxycycline did not affect the ethanol-induced relative mRNA expression of aging markers (**E**) p16 and (**F**) p21. (**G**) Doxycycline ameliorated the relative mRNA expression of GADD45 in ethanol-treated HUVECs. β-actin was used as a loading control. qPCR data are the mean of three technical triplicates, and WB data are the mean of three independent biological triplicates. One-way ANOVA followed by Tukey’s test was used to analyze more than two groups. Error bars represent the SD (*** *p* < 0.001, ** *p* < 0.01, and * *p* < 0.05).

Doxycycline restored the relative protein expression of DNA repair proteins KU70 (Control = 100 ± 10.12, ethanol = 80.38 ± 2.71, doxycycline = 91.53 ± 0.17, ethanol + doxycycline = 99.31 ± 9.67, n = 3, * *p* < 0.05, Figure 4A,B), and KU80 (Control = 100 ± 2.63, ethanol = 83.91 ± 4.28, doxycycline = 93.13 ± 1.22, ethanol + doxycycline = 94.92 ± 5.57, n = 3, ** *p* < 0.01, * *p* < 0.05, Figure 4A,C) in ethanol-treated HUVECs. The reduction in relative protein expression of aging marker lamin b1 after 24 h in ethanol-treated HUVECs was also reversed by doxycycline treatment (Control = 100 ± 7.95, ethanol = 58.93 ± 7.78, doxycycline = 84.27 ± 5.61, ethanol + doxycycline = 82.86 ± 3.56, n = 3, *** *p* < 0.001, ** *p* < 0.01 * *p* < 0.05, Figure 4A,D). Doxycycline did not reduce ethanol-induced relative mRNA expression of aging markers P16 (Control = 1.01 ± 0.14, ethanol = 2.77 ± 0.38, ethanol + doxycycline = 2.88 ± 0.10, n = 3, *** *p* < 0.001, Figure 4E) and P21 (Control = 1.01 ± 0.20, ethanol = 2.50 ± 0.38, ethanol + doxycycline = 2.51 ± 0.19, n = 3, ** *p* < 0.01, Figure 4F). Doxycycline increased the relative mRNA expression of growth arrest and DNA damage-inducible (GADD45) gene in HUVECs exposed to ethanol (Control = 1.00 ± 0.07, ethanol = 0.34 ± 0.09, ethanol + doxycycline = 0.59 ± 0.06, n = 3, *** *p* < 0.001, ** *p* < 0.01, * *p* < 0.05, Figure 4G). Doxycycline rescued the relative protein expression of aging-associated biomarker lamin b1, DNA repair proteins KU70 and KU80, and the relative mRNA expression of GADD45 in ethanol-treated HUVECs.

### 3.6. Doxycycline Attenuated Ethanol-Induced Cellular Senescence and Improved Functional Status of Endothelial Cells

**Figure 5:**
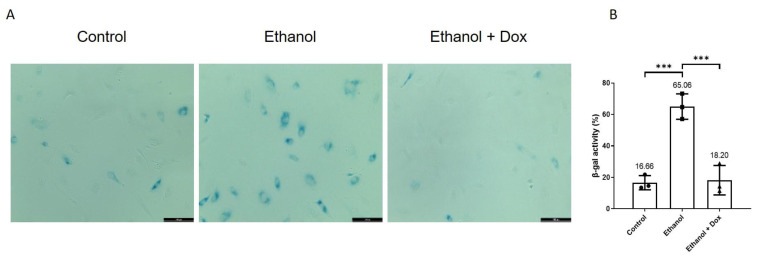
Doxycycline inhibited ethanol-induced cellular senescence in HUVECs. HUVECs were treated with ethanol (400 mM) and combination of ethanol (400 mM) and doxycycline (10 µM) for 4 days. The endothelial cell medium alone was used for control. The medium was changed after two days. (**A**) Images showing increased senescence only in ethanol-treated HUVECs. Scale bar = 100 µm. (**B**) Doxycycline inhibited senescence in HUVECs exposed to ethanol treatment. Data are the mean of three independent experiments. One-way ANOVA followed by Tukey’s test was used to analyze more than two groups. Error bars represent the SD (*** *p* < 0.001).

It has already been known that increased inflammation and telomere shortening lead to cellular senescence [3,4,14]. To investigate if the already observed reduced inflammation and telomere recovery by doxycycline is translated into cellular senescence, β-gal staining was performed. Doxycycline inhibited ethanol-induced senescence in HUVECs, percentage of β-gal positive cells by total cells (Control = 16.66 ± 4.51%, ethanol = 65.06 ± 8.10%, ethanol + doxycycline = 18.20 ± 9.37%, n = 3, *** *p* < 0.001, Figure 5A,B). 

**Figure 6:**
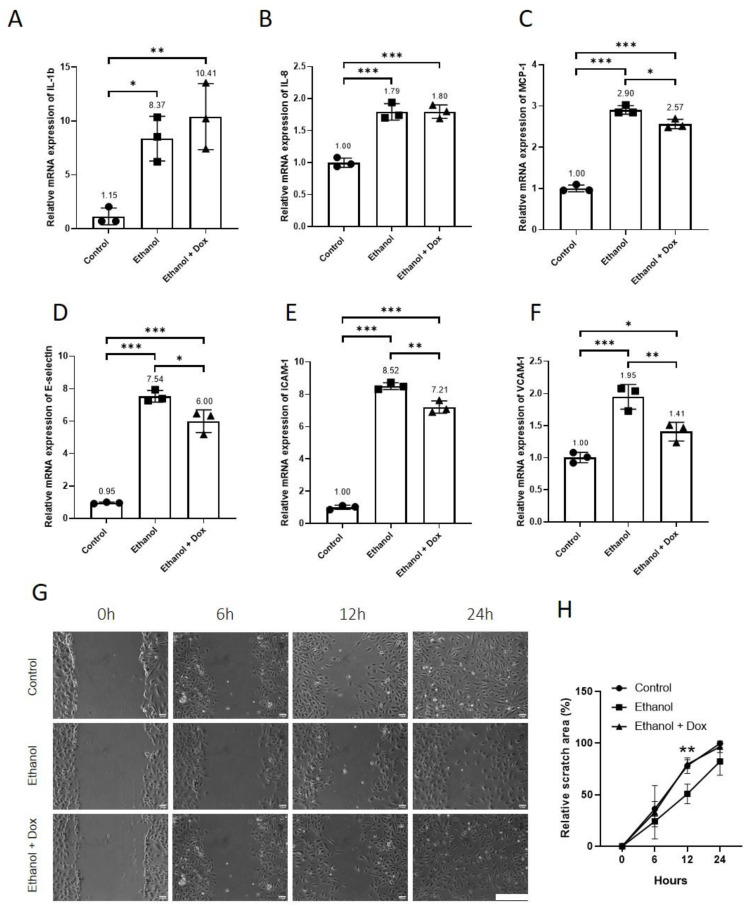
Doxycycline reduced endothelial SASP and dysfunction in ethanol-treated HUVECs. HUVECs were treated with ethanol (400 mM), doxycycline (10 µM), and combination of ethanol (400 mM) and doxycycline (10 µM) for 24 h. The endothelial cell medium alone was used for control. (**A**–**F**) Ethanol treatment increased the relative mRNA expression of L-1β, IL-8, MCP-1, E-selectin ICAM-1, and VCAM-1 after 24 h in HUVECs. Doxycycline did not alter the relative mRNA expression of (**A**) IL-1β and (**B**) IL-8, but it abated the relative mRNA expression of (**C**) MCP-1, (**D**) E-selectin, (**E**) ICAM-1, and (**F**) VCAM-1 after 24 h in ethanol-treated HUVECs. β-actin was used as a housekeeping gene. (**G**) Images showing HUVECs migration under different conditions at different time points after making a scratch using 10 µL pipette tip. Scale bar = 300 µm. (**H**) Quantification analysis showed that doxycycline ameliorated the migration of ethanol-treated HUVECs. qPCR data are the mean of three technical triplicates, and WB data are the mean of three independent biological triplicates. One-way ANOVA followed by Tukey’s test was used to analyze more than two groups. Error bars represent the SD (*** *p* < 0.001, ** *p* < 0.01, and * *p* < 0.05 compared to controls).

As doxycycline inhibited ethanol-induced senescence in HUVECs, we further investigated if doxycycline also reduces SASP in HUVECs exposed to ethanol. The ethanol-treated endothelial cells showed increased relative mRNA expression of pro-inflammatory molecules IL-1β, IL-8, MCP-1, E-selectin, ICAM-1, and VCAM-1 (Figure 6A–F). Doxycycline did not affect the relative mRNA expression of IL-1β (Control = 1.15 ± 0.78, ethanol = 8.37 ± 2.07, ethanol + doxycycline = 10.41 ± 3.06, n = 3, ** *p* < 0.01, * *p* < 0.05 Figure 6A) and IL-8 (Control = 1.00 ± 0.07, ethanol = 1.79 ± 0.13, ethanol + doxycycline = 1.80 ± 0.11, n = 3, *** *p* < 0.001, Figure 6B) in ethanol-treated HUVECs. Doxycycline reduced the relative mRNA expression of MCP-1 (Control = 1.00 ± 0.08, ethanol = 2.90 ± 0.10, ethanol + doxycycline = 2.57 ± 0.11, n = 3, *** *p* < 0.001, * *p* < 0.05, Figure 6C), E-selectin (Control = 0.95 ± 0.05, ethanol = 7.54 ± 0.36, ethanol + doxycycline = 6.00 ± 0.71, n = 3, *** *p* < 0.001, * *p* < 0.05, Figure 6D), ICAM-1 (Control = 1.00 ± 0.13, ethanol = 8.52 ± 0.21, ethanol + doxycycline = 7.21 ± 0.38, n = 3, *** *p* < 0.001, ** *p* < 0.01, Figure 6E) and VCAM-1 (Control = 1.00 ± 0.08, ethanol = 1.95 ± 0.19, ethanol + doxycycline = 1.41 ± 0.15, n = 3, *** *p* < 0.001, ** *p* < 0.01, * *p* < 0.05 Figure 6F) in HUVECs treated with ethanol. The senescent cells are known to show impaired migration [24,25]; therefore, we investigated the effects of ethanol and ethanol combined with doxycycline on endothelial cell migration. Ethanol retarded the migration of HUVECs in wound assay, and doxycycline recovered the migration of HUVECs exposed to ethanol, percentage area covered by migrating endothelial cells at different time points after making a scratch (at 0 h: Control = 0.00 ± 0.01%, ethanol = 0.00 ± 0.00%, ethanol + doxycycline = 0.00 ± 0.00%, after 6 h: Control = 36.27 ± 7.03%, ethanol = 24.20 ± 5.36%, ethanol + doxycycline = 33.00 ± 25.87%, after 12 h: Control = 78.18 ± 7.57%, ethanol = 50.83 ± 9.41%, ethanol + doxycycline = 79.65 ± 4.35%, after 24 h: Control = 99.77 ± 0.23%, ethanol = 82.30 ± 13.40%, ethanol + doxycycline = 96.56 ± 5.64%, n = 3, ** *p* < 0.01, Figure 6G,H). Doxycycline curtailed ethanol-induced endothelial SASP and dysfunction.

**Figure 7:**
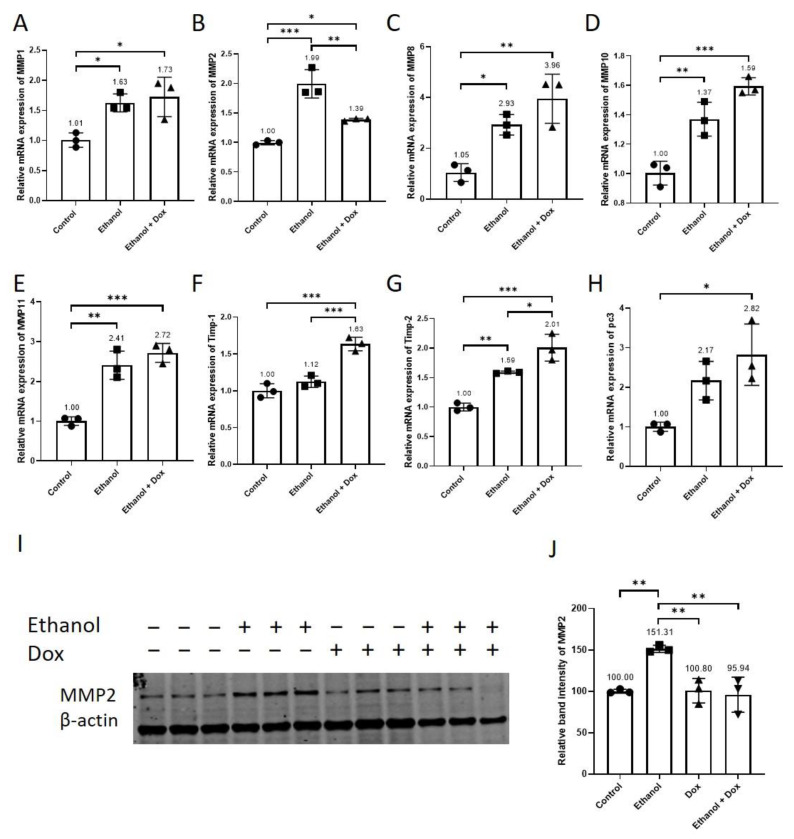
Doxycycline restrained MMP-2 relative mRNA and relative protein expression in ethanol-treated HUVECs. HUVECs were treated with ethanol (400 mM), doxycycline (10 µM), and combination of ethanol (400 mM) and doxycycline (10 µM) for 24 h. The endothelial cell medium alone was used for control. (**A**–**E**,**G**) Ethanol increased the relative mRNA expression of MMP-1, 2, 8, 10, 11, and Timp2 in HUVECs. (**F**–**H**) Doxycycline elevated TIMP-1, TIMP-2, and pc3 relative mRNA expression in HUVECs treated with ethanol. (**B**,**I**,**J**) Doxycycline suppressed ethanol-induced relative mRNA and relative protein expression of MMP2 in HUVECs. β-actin was used as a loading control. qPCR data are the mean of three technical triplicates, and WB data are the mean of three independent biological triplicates. One-way ANOVA followed by Tukey’s test was used to analyze more than two groups. Error bars represent the SD (*** *p* < 0.001, ** *p* < 0.01, and * *p* < 0.05).

The senescent cells release MMPs [8,15], which are known to play an important role in vascular remodeling. Therefore, we investigated the effects of ethanol alone and the combined effect of ethanol and doxycycline on the mRNA expression of MMPs and their inhibitors, namely tissue inhibitor matrix metalloproteinase (TIMP)-1 and TIMP-2. Ethanol increased the relative mRNA expression of MMP-1, MMP-2, MMP-8, MMP-10, MMP-11, and TIMP-2 (Figure 7A–E,G). Doxycycline did not affect the ethanol-induced relative mRNA expression of MMP-1 (Control = 1.01 ± 0.12, ethanol = 1.63 ± 0.15, ethanol + doxycycline = 1.73 ± 0.33, n = 3, * *p* < 0.05, Figure 7A), MMP8 (Control = 1.05 ± 0.35, ethanol = 2.93 ± 0.41, ethanol + doxycycline = 3.96 ± 0.97, n = 3, ** *p* < 0.01, * *p* < 0.05 Figure 7C), MMP10 (Control = 1.00 ± 0.08, ethanol = 1.37 ± 0.12, ethanol + doxycycline = 1.59 ± 0.06, n = 3, *** *p* < 0.001, ** *p* < 0.01 Figure 7D), MMP-11 (Control = 1.00 ± 0.11, ethanol = 2.41 ± 0.35, ethanol + doxycycline = 2.72 ± 0.24, n = 3, *** *p* < 0.001, ** *p* < 0.01 Figure 7E). Doxycycline increased the relative mRNA expression of TIMP-1 (Control = 1.00 ± 0.10, ethanol = 1.12 ± 0.08, ethanol + doxycycline = 1.63 ± 0.09, n = 3, *** *p* < 0.001, Figure 7F), TIMP2 (Control = 1.00 ± 0.07, ethanol = 1.59 ± 0.02, ethanol + doxycycline = 2.01 ± 0.23, n = 3, *** *p* < 0.001, ** *p* < 0.01, * *p* < 0.05, Figure 7G), and procollagenase 3 (pc3) (Control = 1.01 ± 0.12, ethanol = 2.17 ± 0.49, ethanol + doxycycline = 2.83 ± 0.77, n = 3, * *p* < 0.01, Figure 7H) in ethanol-treated HUVECs. Doxycycline reduced the ethanol-induced relative mRNA expression of MMP-2 (Control = 1.00 ± 0.04, ethanol = 1.99 ± 0.24, ethanol + doxycycline = 1.39 ± 0.02, n = 3, *** *p* < 0.001, ** *p* < 0.01, * *p* < 0.05 Figure 7B). The protein analysis showed that doxycycline also impeded the relative protein expression of MMP2 (Control = 100 ± 2.43, ethanol = 151.32 ± 4.32, doxycycline = 100.8 ± 14.76, ethanol + doxycycline = 95.94 ± 21.17, n = 3, ** *p* < 0.01, Figure 7I,J) in ethanol-treated HUVECs. Doxycycline inhibited ethanol-induced relative mRNA and relative protein expression of MMP2 in HUVECs.

## 4. Discussion

In this study, we report that ethanol accelerated molecular aging caused cellular senescence and induced endothelial SASP and dysfunction. Doxycycline dampened ethanol-mediated inflammation and aging in endothelial cells and attenuated cellular dysfunction. Alcohol abuse is one of the potential risk factors associated with cardiovascular diseases. Studies suggest alcohol abuse causes aging [10,11], but the evidence is lacking showing the direct effect of ethanol on cellular aging. In this study, we investigated the effects of ethanol on cellular aging and found that doxycycline is a potential drug to reduce ethanol-induced accelerated aging and cellular senescence in endothelial cells.

We treated HUVECs with two different concentrations of ethanol. Ethanol accelerated aging in HUVECs (Figure 1). The telomere length in ethanol-treated HUVECs was shortened by more than 50% as compared to that in untreated controls (Figure 1A). Constitutive telomere shortening is known to trigger cellular senescence, induce apoptosis and reduce the proliferative capacity of cells [3]. Telomere loss is associated with the pathogenesis of cardiovascular diseases [3,9]. Telomere shortening is linked to vascular aging, and the factors that inhibited telomere shortening also retarded vascular aging [3]. The concentration of doxycycline (10 µM), which alone had no cytotoxic effects and inhibited the cytotoxic effects of ethanol (400 mM) (Section 3.2, Supplementary Figure S1), was used in the experiments. Doxycycline reduced ethanol-accelerated aging in HUVECs (Figure 2A) and inhibited ethanol-induced activation of mTOR (Figure 2B,C). Ethanol activated the mTOR pathway in vivo and in vitro [26,27], and mTOR has been implicated in aging [7,18,19]. Similar to rapamycin inhibition of the mTOR pathway in most cell types [28,29], in our study, doxycycline decreased p-S6 relative protein expression, and it had no effect on 4e-BP1 in ethanol-treated HUVECs (Figure 2B,D,E). Previous studies have shown that rapamycin inhibition of mTOR and depletion of p-S6 delayed the pathogenesis of age-related diseases and increased life span in different animal models [2,7,18,19,30]. Rapamycin inhibition of mTOR reversed age-associated arterial dysfunction and decreased vascular stiffness [2]. In addition to this, mTOR activation has been shown to promote inflammaging via activating NF-kB [6]. Additionally, ethanol has been reported to activate NF-kB in vivo and in vitro [21,22,23]. It is noteworthy that both ethanol and doxycycline reduced mRNA expression of NF-kB, which suggests that ethanol regulates NF-kB differently at transcriptional and posttranslational levels. NF-kB activation has also been implicated in aging [14,15,16,17]. NF-kB activation has been shown to regulate DNA repair protein KU80 [16]. Interestingly, the inhibition of NF-kB delayed and even reversed aging in animal models [14,17]. Our data demonstrate that doxycycline treatment inhibited NF-kB activation (Figure 3A,B), confirming the findings reported previously [12,13]. The constitutive NF-kB activation induced telomere shortening via KU80 dysregulation and increased DNA damage on telomeres [16]. KU80 forms a heterodimer with KU70. KU80/KU70 heterodimer binds to broken DNA and initiates its repair via non-homologous end joining [31,32]. In our study, ethanol decreased the relative protein expression of DNA repair proteins KU70 and KU80, which were recovered after doxycycline treatment. (Figure 4A,C). Animal experiments have shown that the deletion of KU80 and KU70 leads to accelerated aging [33,34]. In a mice study, complete loss of KU80 resulted in premature aging, and loss of a single allele caused accelerated aging in skeletal muscle in mice [35]. The lack of KU70 or KU80 activity resulted in telomere shortening in different mouse cell types [36]. KU80 is essential for human somatic cells [37], and loss of KU80 causes cell death due to massive telomere loss [38]. A decrease in KU70 and KU80 proteins has been reported in senescent cells [39]. The reduced KU70 and KU80 after ethanol treatment can lead to telomere loss and accumulation of DNA damage [36,38]. Moreover, lamin b1 was lost in human and murine cells when DNA damage induced cellular senescence [40]. Doxycycline reduced ethanol-induced cellular senescence and increased the protein expression of lamin b1 (Figure 4D and Figure 5). Loss or reduction in lamin b1 has been reported to increase mTOR activation [41]. Moreover, inhibition of mTOR and its downstream signalling molecule p-S6 reduced cellular senescence [19]. Furthermore, p-mTOR negatively regulates autophagy, which results in the accumulation of damaged proteins and organelles that consequently accelerates the progression of cellular senescence [18,19]. Inhibition of p-mTOR increases autophagy, which protects from proteotoxicity and thus delays cellular senescence [18,19]. In addition to this, p-mTOR inhibition improves mitochondrial function and decreases ROS levels [19], which can provide protection against oxidative stress-induced premature cellular senescence and telomere shortening. Moreover, doxycycline reduced ethanol-induced oxidative stress (Supplementary Figure S2), which suggests doxycycline can provide protection through multiple mechanisms. Multiple studies suggest that telomere shortening and endothelial senescence contribute to vascular aging, and this phenomenon has been implicated in heart failure [2,3]. Senescent cells induced senescence in young cells via a process termed as bystander effect [42]. Senescent cells contribute to age-related diseases by damaging the local environment and promoting tissue remodelling [1], suggesting that doxycycline, by eliminating senescent cells and reducing or inhibiting cellular senescence, can potentially provide protection against age-related vascular diseases [1,5].

Doxycycline did not affect the relative mRNA expression of aging markers P16 and P21 (Figure 4E,F), but it increased the relative mRNA expression of GADD45 (Figure 4G). GADD45 is implicated in stress signalling response, which can lead to cell cycle arrest, DNA repair, cell survival and senescence, or apoptosis, depending on the extent of cellular and DNA damage [43]. NF-kB activation has been shown to promote cellular senescence and aging [6,15,44,45]. Doxycycline inhibited NF-kB activation (Figure 3A,B) [12,13], and inhibition of NF-kB activation delayed cellular senescence and Aging [14,17], which suggests that inflammation can induce premature senescence and aging in endothelial cells [3]. Reciprocally, senescence induces inflammation as senescent cells become more pro-inflammatory and acquire SASP contributing to an amplifying effect [2,5,14,15]. These cells secrete cytokines such as IL-1β, chemokines such as IL-8 and MCP-1, and increase the expression of cell adhesion molecules, namely ICAM-1, VCAM-1 and E-selectin [3,6]. This pro-inflammatory response in senescent and dysfunctional endothelial cells is related to NFκ-B activation [2,8], and inhibition of NFκ-B activation reduces systemic inflammation and improved vasculature [2]. Doxycycline did not affect the mRNA expression of IL-1β and IL-8 (Figure 6A, B), but it inhibited NFκ-B activation (Figure 3A,B) and reduced the mRNA expression of MCP-1, E-selectin, ICAM-1 and VCAM-1 (Figure 6C–F). This direct effect of ethanol on the transcription of cell adhesion molecules in cell systems has not been reported before, although there are reports which suggest chronic consumption of alcohol increased the serum level of E-selectin and ICAM-1 [46]. These molecules promote migration, adhesion and infiltration of leukocytes to vascular endothelium contributing to chronic sterile inflammation [3]. These findings indicate that doxycycline can directly curtail inflammation via suppressing NFκ-B activation, and by reducing the transcription of MCP-1, E-selectin, ICAM-1, and VCAM-1, doxycycline can indirectly reduce sterile inflammation by decreasing the migration and infiltration of inflammatory cells to the arterial wall. 

The migration of endothelial cells is an important process for angiogenesis and repair/healing of damaged tissue. Previous studies have reported that the senescent cells show retarded migration and are not efficient in the repair/healing of damaged tissue [24,25]. Ethanol treatment reduced the migration of HUVECs, and doxycycline significantly recovered the migration of ethanol-treated HUVECs (Figure 6G,H). In the literature, contradictory findings have been reported on the effects of ethanol on cell migration. Wei et al. reported that ethanol promoted the migration of cancerous cells but had no effect on normal cells [47]. Morrow et al. reported that ethanol in low concentration promoted endothelial cell migration [48]. In the same study, they showed that by increasing ethanol concentration, the angiogenic activity in HUVECs decreased [48]. In animal study, ethanol has been shown to impair angiogenesis and wound healing [49]. Doxycycline at higher concentrations (20 µg/mL, 100 µg/mL, and 500 µg/mL) has previously been shown to decrease the migration of epithelial cells and human dermal vascular endothelial cells [50]. The same study showed that doxycycline had a concentration-dependent effect on the migration of these cells [50]. The current findings, together with previous reports, suggest that doxycycline in lower concentrations can promote tissue healing and repair via enhancing endothelial cell migration.

The senescent cells have been shown to release MMPs [8,15], and alcohol consumption has been reported to increase serum levels of MMPs in alcohol abusers [51]. Similar to humans, in animal studies, alcohol elevated MMPs expression in different tissues [52,53,54,55]. In accordance with the previous findings, ethanol increased the relative mRNA expression of different MMPs and the relative protein expression of MMP2 in HUVECs (Figure 7). Doxycycline reduced ethanol-induced relative mRNA and relative protein expression of MMP2 in HUVECs (Figure 7B,I,J). Previously, doxycycline has been shown to curtail MMP2 mRNA expression by reducing its stability and consequently decreasing the protein expression of MMP2 in aortic SMCs [56]. Doxycycline has been reported to impede MMP2 activity [57]. We show that doxycycline increased the relative mRNA expression of TIMP-1 and TIMP-2 in ethanol-treated HUVECs as compared to only ethanol-treated and untreated controls (Figure 7F,G). TIMPs are the most potent inhibitors of MMPs. MMPs are known to play an important role in cardiovascular diseases through different mechanisms, including ECM remodeling, promoting VSMCs migration by cleaving cadherin, and increasing vasoconstriction via cleaving vasoactive precursors such as endothelin-1 and adrenomedullin [8]. MMPs also contribute to inflammation [58]. MMP2 and MMP9 activated IL-1β by cleaving its precursor pro-IL-1β [59]. MMPs had both positive and negative effects on leukocyte recruitment and infiltration via cleaving chemokines [58]. Taken together, these findings suggest that doxycycline treatment can attenuate MMP-2-mediated inflammation and reduce tissue remodeling by directly inhibiting MMP-2 protein expression and indirectly by increasing the expression of its inhibitor TIMP-2.

## 5. Conclusions

Telomere shortening is associated with cardiovascular diseases, but this association does not state that telomere shortening has a causal link to cardiovascular diseases. It is possible that the risk factors that lead to cardiovascular diseases also caused telomere shortening, and the factors that reduced vascular aging also inhibited telomere shortening. Alcohol is a potential risk factor for cardiovascular diseases, and in the present study, we showed that ethanol induced telomere shortening and premature senescence. Doxycycline reduced ethanol-induced inflammaging in HUVECs, possibly by inhibiting activation of the mTOR and NF-kB pathways.

## Data Availability

Data is contained within the article and Supplementary Material.

## Supplementary Materials

The following supporting information can be downloaded at: https://www.mdpi.com/article/10.3390/antiox11122413/s1, Figure S1: CTG assay showing the growth of HUVECs under different conditions. The cells were treated with different concentrations of ethanol (200 mM, 300 mM, 400 mM, 600 mM, and 800 mM,), doxycycline (1 µM, 10 µM, 50 µM, and 100 µM) and combined ethanol (400 mM) and doxycycline (10 µM). The medium was changed after two days. The CTG assay was performed at day 0, day 1, day 2, and day 4.; Figure S2: Immunofluorescence staining for oxidative stress marker 8-OHDG. The cells were treated with ethanol (400 mM), doxycycline (10 µM), and combination of ethanol (400 mM) and doxycycline (10 µM) for two hours. Endothelial medium alone was used for control. (**A**,**B**) Only ethanol-treated HUVECs showed increased oxidative stress. Scale bar = 100 µM, *** *p* < 0.001.; Table S1: Primary and Secondary antibodies.; Table S2: Primer list.
