# Supplementary material for: Doxycycline Attenuated Ethanol-Induced Inflammaging in Endothelial Cells: Implications in Alcohol-Mediated Vascular Diseases
Source: Antioxidants (Basel). 2022 Dec 7;11(12):2413. doi: 10.3390/antiox11122413 (PMC9774758; doi:10.3390/antiox11122413)
Supplement: Supplementary file 1 [file antioxidants-11-02413-s001.zip › antioxidants-1995092-supplementary.pdf]

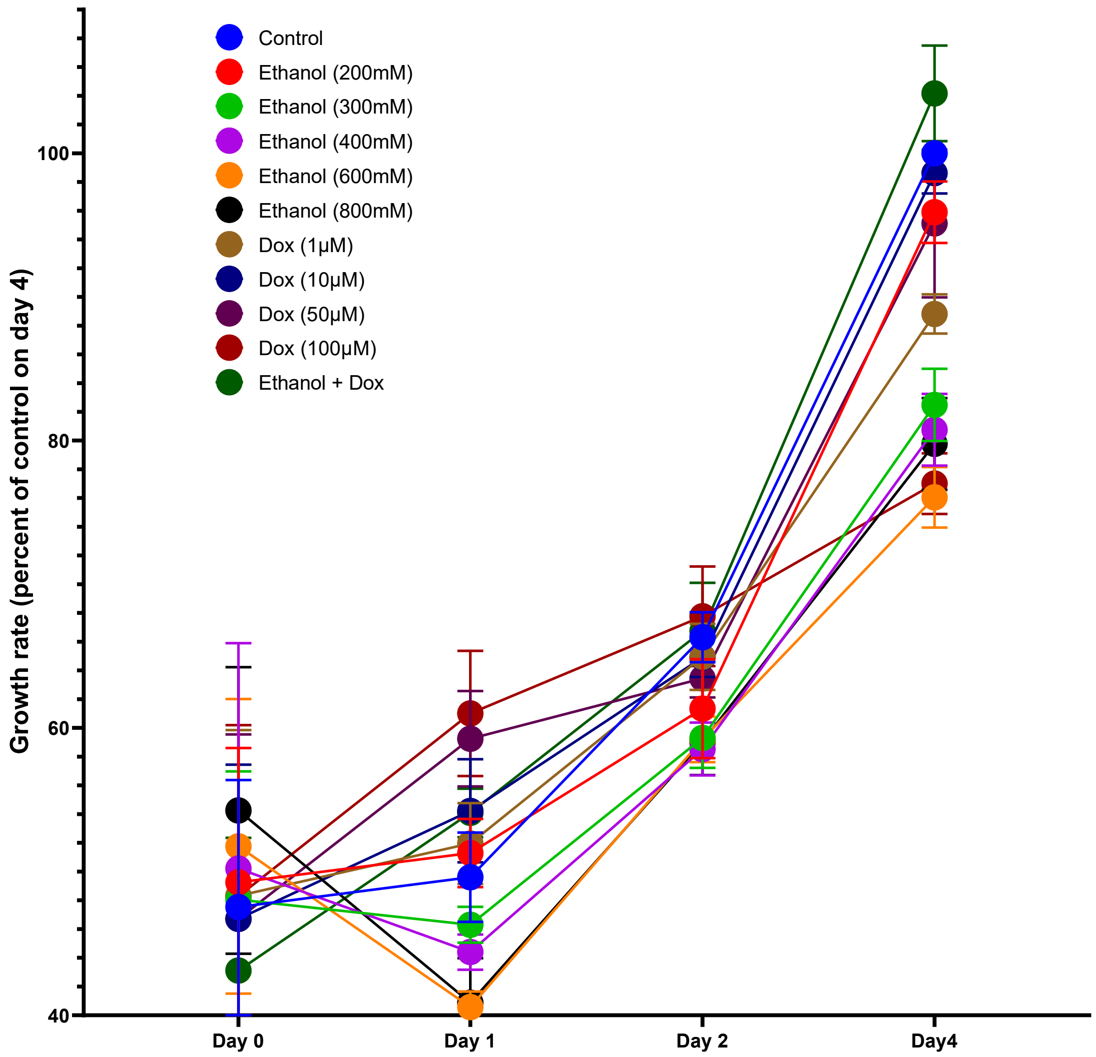

Supplementary Figure S1. CTG assay showing the growth of HUVECs under different conditions. The cells were treated with different concentrations of ethanol (200 mM, 300 mM, 400 mM, 600 mM, 800 mM,), doxycycline (1 µM, 10 µM, 50 µM, 100 µM) and combined ethanol (400 mM) and doxycycline (10 µM). The medium was changed after two days. The CTG assay was performed at day 0, day 1, day 2 and day 4.

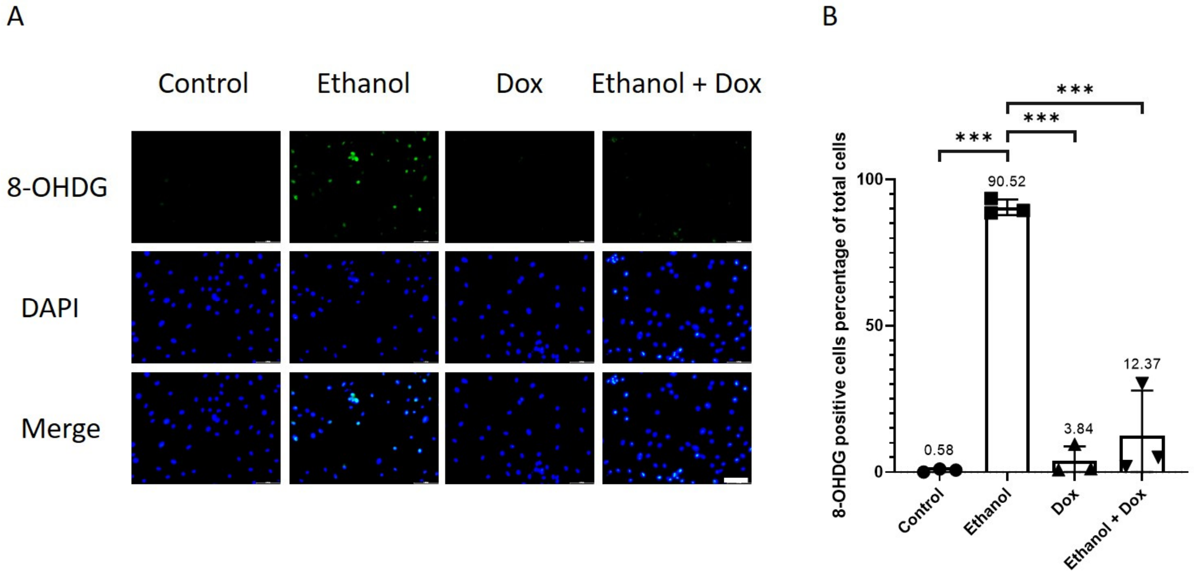

Supplementary Figure S2. Immunofluorescence staining for oxidative stress marker 8-OHDG. The cells were treated with ethanol (400 mM), doxycycline (10 μM) and combination of ethanol (400 mM) and doxycycline (10 μ M) for two hours. Endothelial medium alone was used for control. (A, B) Only ethanol-treated HUVECs showed increased oxidative stress. Scale bar = 100 μM, p*** < 0.001.

Table S1. Primary and Secondary antibodies

Antibody	MW(kDa)	Brand	Catalog Number	Concentration
Lamin-b1	66	Abcam	Ab16048	1:1000
KU70	70	Cell Signaling	4588s	1:1000
KU80	86	Cell Signaling	2753	1:1000
NFKB	65	Abcam	ab16502	1:1000
p-NFKB	65	Cell Signaling	3033S	1:1000
MMP-2	70	Invitrogen	436000	2 µg/mL
β-actin	45	Cell Signaling	4970S	1:1000
p-mTOR	289	Cell Signaling	2971S	1:1000
P-4ebp1	15-20	Cell Signaling	9451T	1:1000
p-S6	32	Cell Signaling	2215S	1:1000
S6	32	Cell Signaling	2217S	1:1000
IRDye 800CW		LiCor	926-32211	1:10,000
IRDye 680RD		LiCor	926-68072	1:10,000
8-OHDG		BIOSS	BSS-BS-1278R	1:500
Alexa Fluor 488		abcam	ab150077	1:1000

Table S2. Primer list

Target gene	Gene Accession Number	Sense 5' -3'	Antisense 5' -3'
P16	NM_000077, NM_058197, NM_001195132	CAACGCACCGAATAGTTACG	AGCACCACCAGCGTGTC
P21	NM_001374511, NM_001220777, NM_001374510, NM_001374512, NM_001374513, NM_001291549, NM_078467, NM_001374509, NM_001220778, NM_000389	GACACCACTGGAGGGTGACT	CAGGTCCACATGGTCTTCCT
ICAM-1	NM_000201	CACAGTCACCTATGGCAACGA	TGGCTTCGTCAGAATCACGTT
VCAM-1	NM_080682, NM_001199834, NM_001078	AGTGGTGGCCTCCTGAATGG	CTGTGTCTCCTGTCTCCGCT
TEL		CGGTTTGTTTGGGTTTGGGTTTGGGTTTGGGTTTGGGTT	GGCTTGCCTTACCCTTACCCTTACCCTTACCCTTACCCT
IFNB1	NC_000009	GGTTACCTCCGAAACTGAAGA	CCTTTCATATGCAGTACATTAGCC
IL-8	NM_001354840, NM_000584	TGCCAAGGAGTGCTAAAG	CTCCACAACCCTCTGCAC
MCP-1	NM_002982	CACCAATAGGAAGATCTCAGTGC	TGAGTGTTCAAGTCTTCGGAGTT
MMP1	NM_001145938, NM_002421	CAGAGATGAAGTCCGGTTTTTC	GGGGTATCCGTGTAGCACAT
MMP2	NM_001302510, NM_001302509,	ATAACCTGGATGCCGTCGT	AGGCACCCTTGAAGAAGTAGC

	NM_001127891, NM_004530, NM_001302508		
MMP8	NM_001304442, NM_001304441, NM_002424	TGGGGCTCGCTCACTCCTC	ATCAAATGTCAAACTGGGGTCAC
MMP10	NM_002425	CACAGTTTGGCTCATGCCTA	TGCCATTCACATCATCTTGC
MMP11	NM_005940	CCGCAACCGACAGAAGAGG	ATCGCTCCATACCTTTAGGGC
TIMP1	NM_003254	TGGCTTCTGGCATCCTGTTGTTG	CGCTGGTATAAGGTGGTCTGGTTG
TIMP2	NM_003255	GAATCGGTGAGGTCCTGTCCTGA	CCTGCACACAAGCCCGGATAAA
IL-1b	NM_000576	AGATGATAAGCCCACTCTACAG	ACATTCAGCACAGGACTCTC
Pro-Collagenase3	NM_000090	CTGGACCCCAGGGTCTTC	GACCATCTGATCCAGGGTTTC
E-selectin	NM_000450	CAAGAAGAAGCTTGCCCTATG	ACTTGAGTCCACTGAAGCCA
NF-κB	NM_001404662, NM_001404661, NM_001404658, NM_001404663, NM_001404657, NM_001243985, NM_001145138, NM_001243984, NM_021975,	TCAAGATCTGCCGAGTGAAC	CCTCTTTCTGCACCTTGTCA
